# AKT-mTOR signaling modulates the dynamics of IRE1 RNAse activity by regulating ER-mitochondria contacts

**DOI:** 10.1038/s41598-017-16662-1

**Published:** 2017-11-28

**Authors:** Miguel Sanchez-Alvarez, Miguel Angel del Pozo, Chris Bakal

**Affiliations:** 1 Dynamical Cell Systems Team, Division of Cancer Biology, The Institute of Cancer Research-Chester Beatty laboratories, 237 Fulham Rd, SW3 6JB London, United Kingdom; 2 Mechanoadaptation and Caveolae Biology Lab, Area of Cell and Developmental Biology, National Centre for Cardiovascular Research (CNIC), c/Melchor Fernandez Almagro, 8, CP, 28029 Madrid, Spain

## Abstract

Inositol Requiring Enzyme-1 (IRE1) is the most conserved transducer of the Unfolded Protein Response (UPR), a surveillance mechanism that ensures homeostasis of the endoplasmic reticulum (ER) in eukaryotes. IRE1 activation orchestrates adaptive responses, including lipid anabolism, metabolic reprogramming, increases in protein folding competency, and ER expansion/remodeling. However, we still know surprisingly little regarding the principles by which this ER transducer is *deactivated* upon ER stress clearance. Here we show that Protein Kinase B-mechanistic Target of Rapamycin (PKB/AKT-mTOR) signaling controls the dynamics of IRE1 deactivation by regulating ER-mitochondria physical contacts and the autophosphorylation state of IRE1. AKT-mTOR-mediated attenuation of IRE1 activity is important for ER remodelling dynamics and cell survival in the face of recursive, transient ER stress. Our observations suggest that IRE1 attenuation is an integral component of anabolic programmes regulated by AKT-mTOR. We suggest that AKT-mTOR activity is part of a ‘timing mechanism’ to deactivate IRE1 immediately following engagement of the UPR, in order to limit prolonged IRE1 RNAse activity that could lead to damaging inflammation or apoptosis.

## Introduction

Eukaryotes have evolved a complex signaling system, termed the Unfolded Protein Response (UPR), to continuously gauge protein folding competency and membrane integrity in the lumen of the endoplasmic reticulum (ER), and engage programmes that promote ER homeostasis. In higher eukaryotes, the UPR consists of three main branches. During ER stress, the eIF2alpha kinase EIF2AK3/PERK branch functions primarily to attenuate mRNA translation in the cell - thus reducing ER client load^1^. Furthermore, during ER stress endopeptidases process Activation Transcription Factor-6 (ATF6) into a transcription factor controlling the expression of ER function regulators such as chaperones and red/ox modulators^2^. Inositol Requiring Enzyme-1 (IRE1/ERN1) is an unconventional transmembrane serine/threonine kinase bearing a C-terminal RNAse module, which is conserved across eukaryotic subtaxa, and its alpha isoform is the only one essential for embryonic viability in mammals (from hereon, references to mammalian IRE1 will refer to IRE1α)^3^. When activated by binding of unfolded proteins; loss of the repressive interaction with the Binding Immunoglobulin Protein (BiP/Grp78) chaperone; or altered ER membrane properties^4–6^, IRE1 catalyzes the extranuclear processing of the X-box Binding Protein-1 (XBP1) transcript^7^. This unconventional splicing changes the open reading frame (ORF) of *xbp1* mRNA, which is then translated into an active transcription factor orchestrating the expression of regulators that ultimately enhance the functional capacity of the ER^8^. Above certain activation thresholds, IRE1 also targets mRNA subsets for degradation (RIDD: Regulated IRE1-Dependent Degradation)^9^.

While a transient UPR promotes ER homeostasis, its sustained or excessive activation can lead to chronic inflammation or apoptosis^3,8,10–12^. Detailed models exist describing the physical clustering, conformational changes and autophosphorylation events that take place upon its activation^4,5,13–16^; however knowledge regarding the *deactivation* of IRE1 upon ER stress clearance is more limited. Studies in yeast have shown that IRE1 deactivation is an active process, and does not simply follow the progressive decrease in misfolded ER luminal proteins. Specifically, transient phosphorylation and subsequent dephosphorylation of residues within, or adjacent to, the kinase activation loop (KAL) of IRE1, are required for the attenuation of its RNAse activity. Mutation of these residues in yeast yields strains with reduced resilience to sustained stress^17,18^, but the relevance of the observations to mammalian cells and tissues remains as yet unproven^19,20^. Additional structural elements may also be involved in tuning IRE1 activity; for example, cysteine red/ox or poly-ADP ribosylation can modulate IRE1 RNAse output^21,22^. The spatial localization of IRE1 activity and its relationship with adjacent organelles, such as the mitochondria, seems to be also relevant in defining its output^6,23–25^.

One means by which the activity of the UPR could receive such multivariate inputs, is via mitochondria-associated membranes (MAMs)- which serve as platforms where signaling systems integrate environmental and cellular conditions, and in turn coordinate diverse cellular processes. MAMs are specialized ER subdomains physically linked to adjacent mitochondria delineating points of extensive physical and functional ER-mitochondria “contact” – or regions where ER and mitochondria are in close juxtaposition where they are separated by only 9–30 nm. One key role of MAMs is to facilitate the trafficking of lipids and calcium between both organelles, which might otherwise be difficult to traffic through the cytosol (reviewed in^26^). Interestingly, regulators of growth such as AKT appear to be enriched at MAMs, and insulin/AKT activity drives the formation of further ER-mitochondrial contacts^27–29^, suggesting that ER-mitochondrial contacts can be formed in response to conditions favorable for growth, facilitating anabolic processes. The UPR transducers IRE1 and PERK have been reported to localize to MAMs^23,30^, but the functional significance of such partition is very poorly understood.

Here, we report that the AKT-mTOR signaling axis promotes the attenuation of IRE1 splicing activity in higher eukaryotes. AKT-mTOR inhibition during ER stress, results in prolonged IRE1 spatial clustering, and splicing activity- even when ER function has been restored to near-normal levels. IRE1 autophosphorylation sites are not strictly required for IRE1 RNAse activity triggering, but are necessary for AKT-mTOR-dependent attenuation of IRE1 RNAse activity. Prolonging IRE1 activation by transiently inhibiting the AKT-mTOR axis leads to increased survival in the face of recursive ER stress challenges. We found that the AKT-mTOR axis inhibits IRE1 activity by promoting ER-mitochondria contacts. We propose that the AKT-mTOR pathway suppresses IRE1 activity to ensure restoration of cell growth and proliferation in a coordinated manner with organelle dynamics reflected by changes in ER-mitochondria contacts.

## Results

### AKT-mTOR inhibition during ER stress recovery delays IRE1 attenuation in a S6K -and ER stress clearance-independent manner

We have previously observed that sustained inhibition of mTOR results in increased IRE1 RNAse activation^31,32^, and thus, in genetic terms mTOR is an IRE1 suppressor. The mechanisms behind the suppression are in part due to the inability of mTOR-inhibited cells to activate Sterol Response Element Binding Proteins (SREBPs), and generate lipids needed for ER homeostasis; which results in ER stress, and promotes the activation of IRE1 RNAse even in the absence of exogenous stressors^31,32^. But we aimed to determine if mTOR could also potentially suppress IRE1 activity in a more direct fashion - for example by *downregulating* IRE1 RNAse activity once the UPR has been engaged.

**Figure 1:**
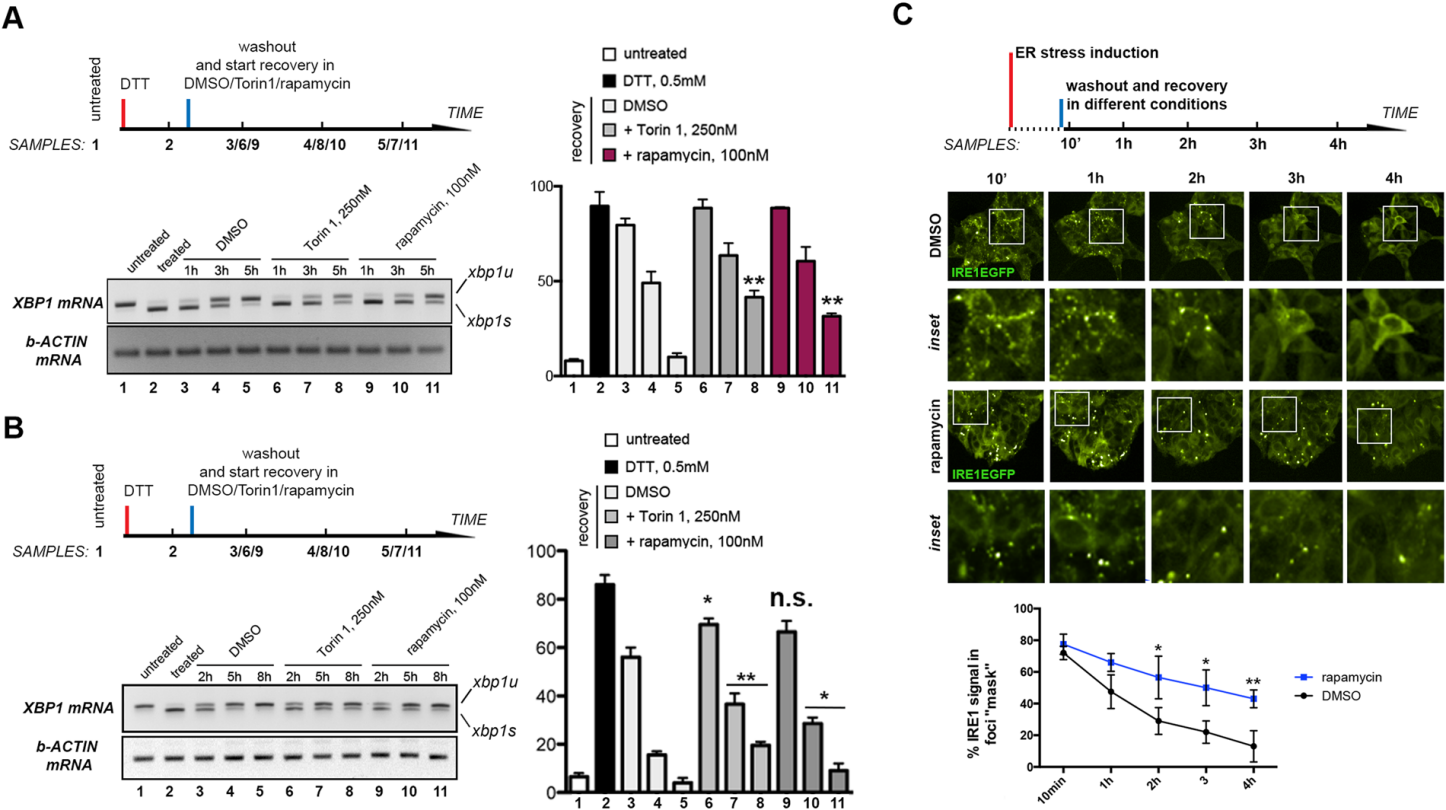
mTOR signaling attenuates IRE1 RNAse during ER stress recovery. (**A**) S2R+ cells were treated as indicated. Total RNA was subsequently extracted for semiquantitative RT-PCR analysis of XBP1 mRNA species (*xbp1s:* spliced XBP1 mRNA signal; *xbp1u:* unspliced XBP1 mRNA signal). Graphs represent four independent biological replicates. Statistical significance was calculated as t-Student *p* value as compared with the corresponding recovery time points of control cells; *n.s* non-significant; *p < 0.05 **p < 0.02. (**B**) MCF10A cells were treated as indicated. Total RNA was subsequently extracted for semiquantitative RT-PCR analysis of XBP1 mRNA species (*xbp1s:* spliced XBP1 mRNA signal; *xbp1u:* unspliced XBP1 mRNA signal). Graphs represent four independent biological replicates. Statistical significance was calculated as t-Student *p* value as compared with the corresponding recovery time points of control cells; *n.s* non-significant; *p < 0.05 **p < 0.02. (**C**) IRE1-EGFP2v 293T cell clone samples were treated for 4 h with tunicamycin (1 μg/ml). Subsequently cells were washed three times with fresh complete medium, and allowed to recover while performing automated imaging and image analysis in a conditioned chamber for the indicated time points. Blow-ups of the indicated ROIs across treatments are shown. Relative intensity distribution as related to bright IRE1EGFP clusters is indicated, from 6 independent experimental replicates.

To gain insight into whether mTOR regulates the dynamics of IRE1 deactivation, we developed assays to acutely inhibit mTOR specifically during recovery from ER stress. Firstly, we used small-molecules to block mTOR activity immediately after exposure to and removal of a chemical ER stressor. To induce the UPR we first used the fast-acting, universal ER stressor dithiothreithol (DTT), which provokes a brisk reduction of the oxidizing environment of the ER lumen, and causes accumulation of incompletely folded proteins^33^. mTOR inhibition either using the TOR kinase competitor Torin1^34^ or the mTOR Complex 1-specific inhibitor rapamycin^35^ significantly delayed the attenuation of IRE1 splicing activity, as assessed by semiquantitative RT-PCR analysis of XBP1 mRNA species in *Drosophila* and human cells (Fig. 1A and B, respectively). Sustained RNAi knockdown of the mTOR kinase also significantly delayed the attenuation of IRE1 splicing (Figure S1A). Conversely, activation of mTOR signaling by insulin stimulation accelerated the attenuation of IRE1 RNAse activity upon removal of the source of ER stress (Figure S1B). Thus, mTOR activity attenuates the IRE1 branch of UPR signaling during recovery from ER stress in a conserved manner. Notably, different cell lines exhibited distinct sensitivity to the influence of mTOR inhibition during IRE1 shutdown. For example, IRE1 RNAase activity in HeLa cells exhbited low sensitivity to mTOR inhibition (Figure S1C).

Spatial clustering of IRE1 correlates with IRE1 RNase activation (Figure S1B and C)^14,15,36^. To examine whether mTOR activity affects IRE1 clustering upon engagement of the UPR, we established HEK293T clonal lines expressing an EGFP-tagged version of IRE1 to monitor IRE1 clustering in living human cells^14,36^. Attenuation of IRE1 RNAse activity following washout of tunicamycin correlated well with the dissolution of IRE1 clusters (Figure S1D and E). Importantly, mTOR inhibition after removal of ER stress significantly delayed IRE1 cluster dissolution (Fig. 1C). These observations support the notion that mTOR regulates UPR dynamics by favouring the deactivation of IRE1 itself. Of note, ATF6 endomembrane cleavage was also attenuated upon removal of ER stress source, but this attenuation was insensitive to mTOR inhibition (Figure S2A). The dynamics of eIF2alpha phosphorylation (PERK-dependent) were neither significantly affected by similar treatment regimes (Figure S2B). Thus, AKT-mTOR signaling specifically regulates the attenuation of the IRE1 branch of the UPR.

**Figure 2:**
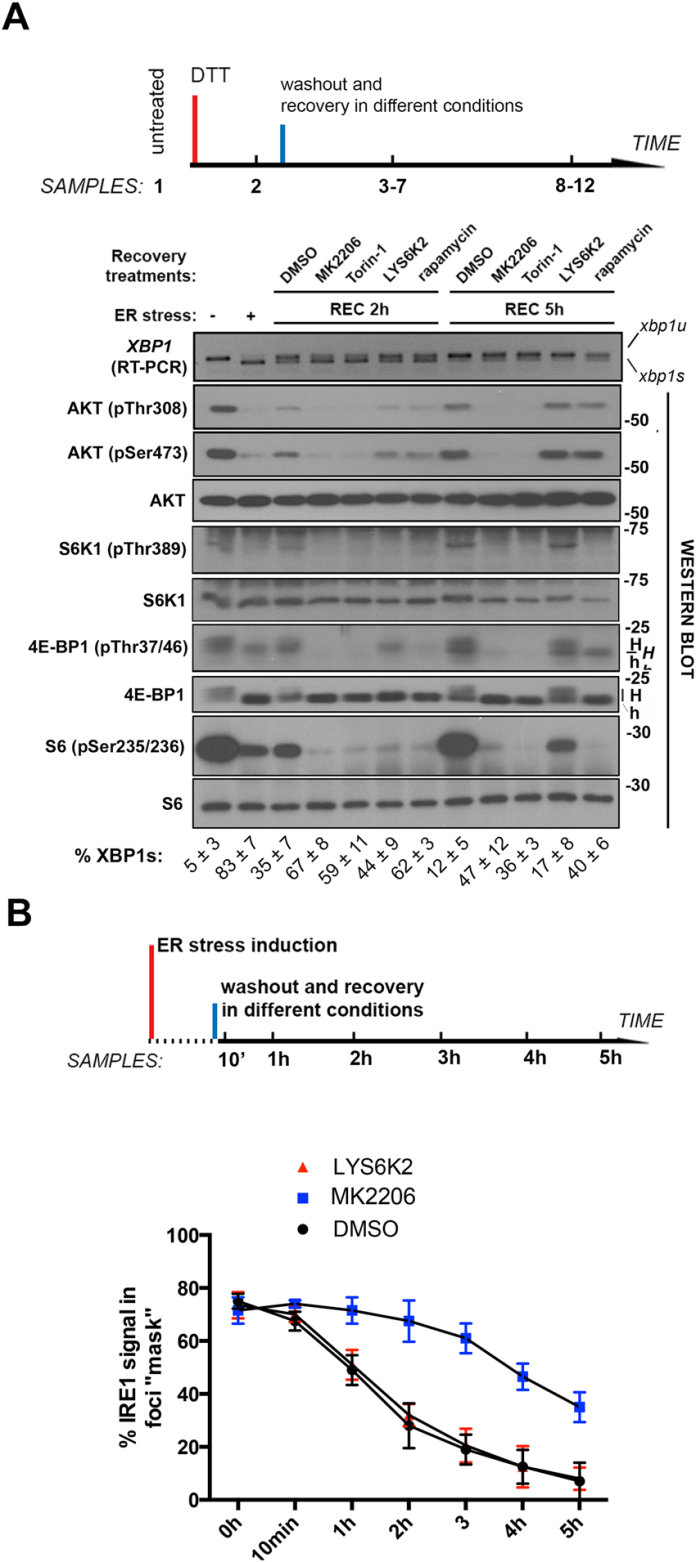
mTORC1-dependent IRE1 attenuation is AKT-dependent and S6K-independent. (**A**) Analysis of the effect of different kinase inhibitors on the dynamics of IRE1 attenuation during ER stress recovery. Inhibitors tested were (in the same order as indicated in the figure): MK2206 (AKT inhibitor, 10 μM; lanes 4 and 9); Torin1 (TOR kinase inhibitor, 500 nM; lanes 5 and 10); LYS6K2 (S6 kinase inhibitor, 500 nM; lanes 6 and 11); and rapamycin (mTORC1 complex inhibitor, 100 nM; lanes 7 and 12). MCF10A cells were treated as specified, and total RNA (XBP1 species analysis, top panel; *xbp1u:* unprocessed species; *xbp1s:* spliced species) and total protein lysates (western blot analysis as labeled) were harvested. Numbers on the right indicate approximate molecular weight standards; numbers in the bottom indicate lane numbers. *H*: hyperphosphorylated species of 4EBP1; *h*: hypophosphorylated forms of 4EBP1. XBP1 u/s ratio quantitation (*lowest row of numbers*) was derived from 4 independent biological replicates. (**B**) IRE1-EGFP2v clone cells were exposed for 4 h to tunicamycin (1 μg/ml), washed out and allowed to recover under the indicated treatments, while simultaneously imaging cells in a conditioned chambered microscope. Relative intensity distribution as related to bright IRE1EGFP clusters is indicated, from 6 independent experimental replicates.

We sought to further characterize signals that act upstream and downstream of mTOR-dependent regulation of IRE1 RNAse activity during engagement of the UPR and recovery from ER stress. Acute ER stress in normal human epithelial cells leads to inhibition of the AKT-mTOR pathway as assessed by western blot analysis of AKT (Ser serine residues 308 and 473) and substrates downstream AKT-mTORC1: S6 (Ser residues 235 and 236) and 4E binding protein 1 (4EBP1; residues Thr 37 and 46) (Fig. 2A, lane 2)^37^. Upon washout of tunicamycin, attenuation of IRE1 splicing coincides with the reactivation of AKT-mTOR signaling (Fig. 2A, lanes 3 and 8). As expected, addition of either Torin1 or rapamycin resulted in deficient reactivation of mTOR signaling upon tunicamycin washout (Fig. 2A, lanes 5 and 10, and 7 and 12, respectively). We then tested whether inhibitors of AKT (MK2206) or the S6 kinase (S6K) (LYS6K2) affect IRE1 activation following its engagement. Importantly, AKT inhibition, but not S6K blockade, also prolongs IRE1 RNAse activity following engagement of the UPR (Fig. 2A, compare lanes 4 and 9 (AKT inhibitor) with lanes 6 and 11 (S6K inhibitor)). We also tested the effect of these drugs on the spatial clustering dynamics of IRE1-EGFP. We found that the exposure of cells to the AKT inhibitor, but not the S6K inhibitor, delays cluster disassembly (Fig. 2B).

**Figure 3:**
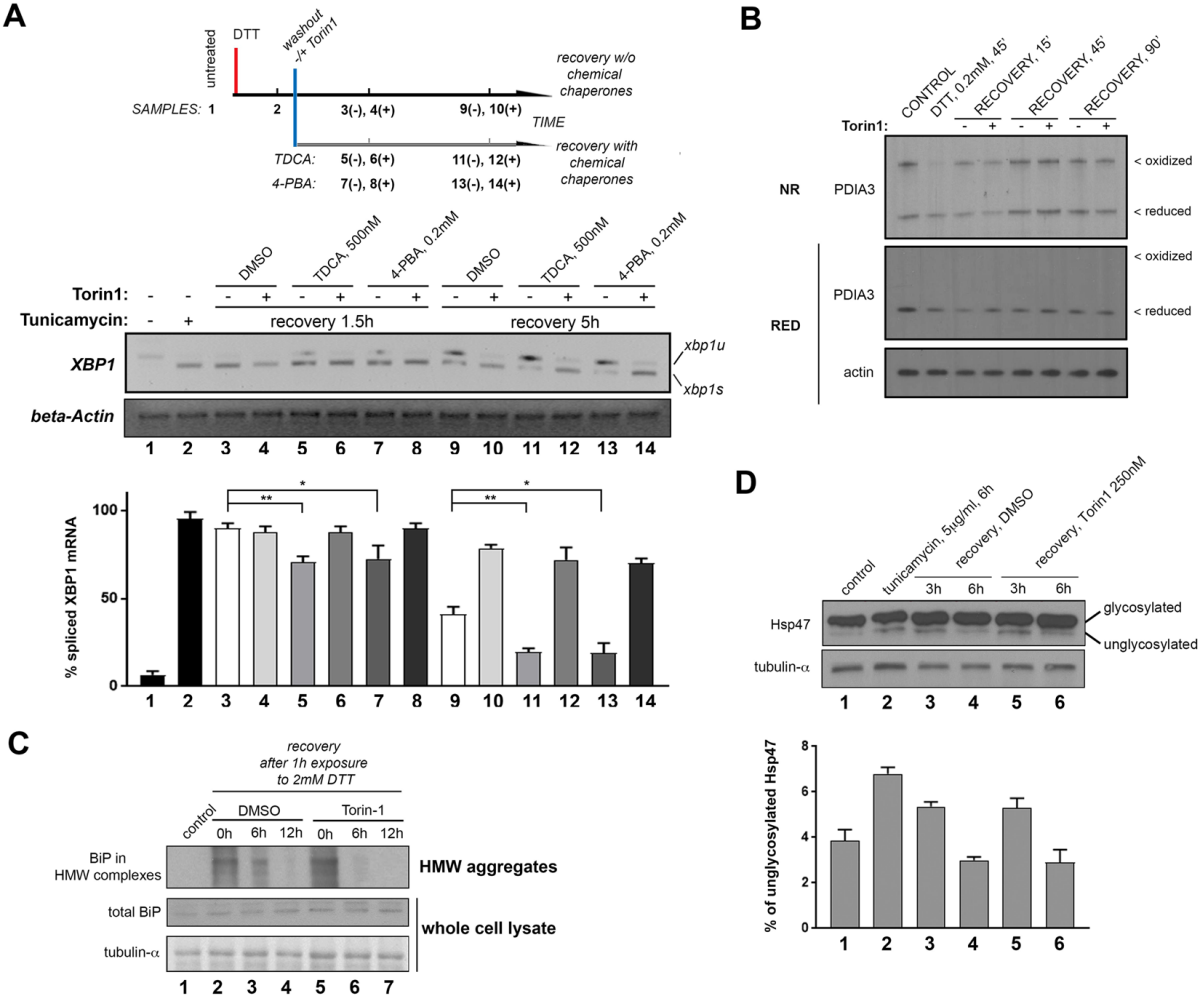
Inhibition of mTOR1 does not prolong IRE1 RNAse activity due to increases in ER stress. (**A**) Chemical chaperones TDCA or 4-PBA are not capable of reverting the delay in IRE1 attenuation induced by TOR kinase inhibition during ER stress recovery. TDCA: tauroursodeoxycholic acid; 4-PBA: 4-phenylbutiric acid. Cells were treated as indicated, and total RNA was extracted for RT-PCR analysis of XBP1 mRNA species. Data was derived from four experimental replicates. (**B**) Non-reducing SDS-PAGE analysis of endogenous species of PDIA3. MCF10A were treated as indicated (Torin1: 500 nM) and immediately alkylated and lysed in non-reducing sample buffer and analyzed by western blot analysis. Equal amounts of unalkylated sample were lysed in DTT-containing sample buffer and run in parallel for total PDIA3 levels. Oxidized species and reduced species are indicated in the upper panel (NR: non-reducing SDS-PAGE). (**C**) High molecular weight (HMW) aggregates from MCF10A cells either left untreated, or exposed to 2 mM DTT for 1 h and then allowed to recover in fresh media with the indicated treatments for the indicated times, were isolated by sucrose cushion centrifugation, solubilized in 8 M urea-sample buffer and analyzed by western blot (38) [top panel]. Unprocessed extracts were analyzed in parallel [middle and bottom panels]. (**D**) Whole cell lysates from MCF10A cells treated as indicated were resolved through gradient SDS-PAGE gels, and analyzed by western blot for species of the Hsp47 collagen chaperone. Glycosylated (top band) and hypoglycosylated (lower band) species are indicated in the corresponding western blot. Densitometry data is shown in the accompanying graph for three biological replicates.

The fact that AKT-mTOR inhibition specifically prolongs IRE1 RNAse activation, but not the dynamics of ATF6 cleavage or eIF2alpha phosphorylation, suggested that AKT-mTOR regulates the spatial clustering and/or posttranslational modification of IRE1, instead of affecting the competency of the ER to clear the source of ER stress. To rule out the possibility that mTOR and AKT inhibition could be prolonging IRE1 RNAse activation by reducing the client peptide folding capacity of the ER, we decided to test whether the pharmacological enhancement of peptide folding capacity in AKT-mTOR inhibited cells could restore the period required for IRE1 RNAse deactivation to a duration comparable to that observed in wild-type cells. We assessed the influence on IRE1 attenuation across recovery conditions of two different exogenous chemical chaperones, 4-phenylbutirate (4-PBA) or tauroursodeoxycholic acid (TDCA). These chemical chaperones promote the clearance of luminal misfolded polypeptides both *in vitro* and *in vivo*^38^. Accordingly, in MCF10A cells after ER stress challenge, both compounds slightly attenuate IRE1-dependent XBP1 mRNA splicing (Fig. 3A, compare lanes 3/5/7 and 9/11/13). However, in the presence of the mTOR kinase inhibitor Torin1, IRE1 RNAse activity is prolonged, and neither chemical chaperone is capable of altering these dynamics (Fig. 3A, compare lanes 4/6/8 and 10/12/14).

Next, we determined whether mTOR inhibition after removal of ER stress affects the restoration of ER luminal folding capacity as assessed by the ratio of reduced-to-oxidized pools of protein disulfide isomerase 3 (PDI3A) following treatment with DTT. mTOR kinase inhibition did not affect redox conditions following the recovery of cells from DTT as compared with cells recovered in normal conditions (Fig. 3B). Furthermore, despite the observed delay in IRE1 attenuation, cells exposed to mTOR inhibition during ER stress recovery did not exhibit a delay in the rate of clearance of high molecular weight aggregates containing the luminal chaperone BiP^39^ (Fig. 3C). Finally, we did not find differences in terms of the ability of cells to re-establish glycosylation activity upon washout of the reversible N-glycosyl transferase inhibitor tunicamycin (TM) across recovery conditions, as assessed by relative glycosylated and unglycosylated endogenous pools of the heat shock protein 47 (Hsp47) (Fig. 3D). Thus, AKT-mTOR signaling blunts IRE1 RNAse activity directly during ER stress recovery, and the prolonged IRE1 RNAse activity observed in mTOR-inhibited cells is not derived from deficient clearance of misfolded and/or unprocessed peptides in the ER. In fact, insoluble protein aggregates are cleared *faster* in mTOR-inhibited cells- perhaps as a consequence of extended IRE1 activation itself, or parallel mechanisms such as ERAD (Fig. 3C, compare lanes 3 vs 6). Thus inhibition of AKT-mTOR signaling during ER stress leads to enhanced ER capacity.

### AKT/mTOR-dependent IRE1 attenuation modulates ER architecture dynamics

**Figure 4:**
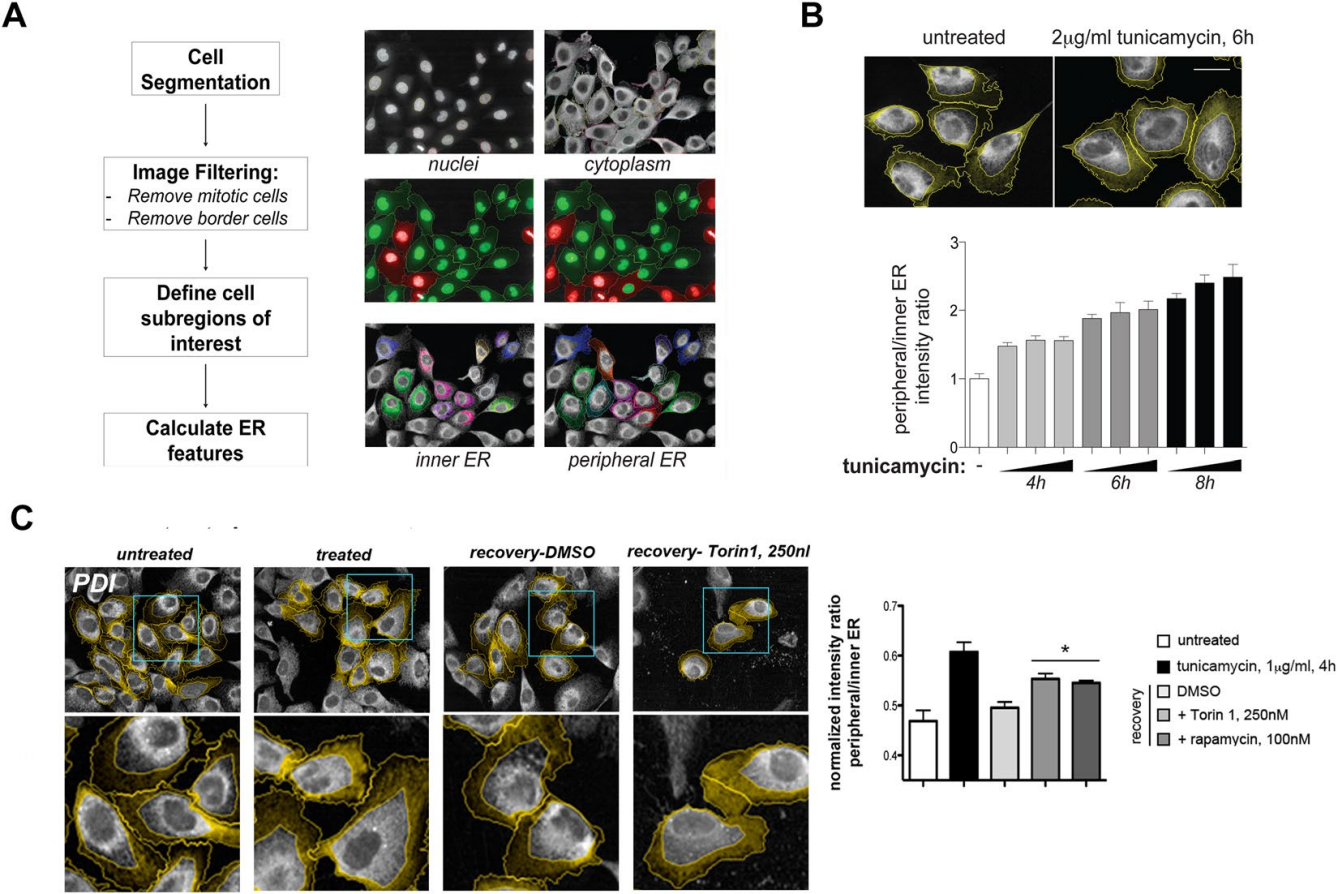
mTOR-dependent IRE1 attenuation of RNAse activity has a significant impact on ER remodelling dynamics. (**A**) A quantitative imaging approach to estimate the extent of ER remodelling in human MCF10A epithelial cells. Main image processing and analysis steps and segmentation examples are shown in the schematic depiction. (**B** and **C**) A measurement of relative ER density distribution comparing inner and peripheral regions of the cell captures ER expansion in tunicamycin-treated MCF10A cells, as compared with mock-treated cells. Four experimental replicates, containing ~1200 cells each, were imaged and quantified as indicated. Wash-out of the ER stressor leads to reaccomodation of the ER towards perinuclear regions over time [**C**; lower left panel and graph]. mTOR inhibition immediately after ER stressor washout however delays this reaccomodation. Lower panel row shows magnified images of the selected ROIs.

One of the key roles of the IRE1 axis is to promote changes in ER volume and shape. Such remodeling is thought to increase the net capacity of the ER to fold, modify, and transport newly synthesized peptides^31,40,41^. ER expansion can be measured by quantitative single-cell image analysis (Fig. 4A^31^), as delimiting peripheral and inner cell areas based on constant relative percentages of cell perimeter, allows for reliable estimation of relative subcellular ER distribution. Peripheral ER is significantly increased upon exposure to tunicamycin (Fig. 4B). However, these changes in ER morphology are transient as ER function is restored - which can also be quantified. In fact we observe a correlation in the dynamics of restoration of ER architecture and those of IRE1 RNAse activity attenuation (Fig. 4C). We thus sought to determine whether inhibition of mTOR signaling, which prolongs IRE1 signaling even when ER stress is resolved, also leads to prolonged ER expansion. Exposure to either Torin1 or rapamycin specifically after washout of ER stressor extends the period over which ER expansion occurs, which is significantly longer than the expansion observed in cells with functional TOR activity (Fig. 4C). These observations strongly suggest that the prolonged activation of the UPR in the absence of ER stress upon AKT-mTOR inhibition has a significant impact not only on IRE1 RNAse activity, but also on downstream effects of IRE1 activation such as ER expansion. Moreover, this suggests that activation of AKT-mTOR during periods of anabolic growth will limit ER expansion, even though the client load of the ER increases.

### Prolonged IRE1 RNAse activity following inhibition of AKT-mTOR increases long-term fitness

**Figure 5:**
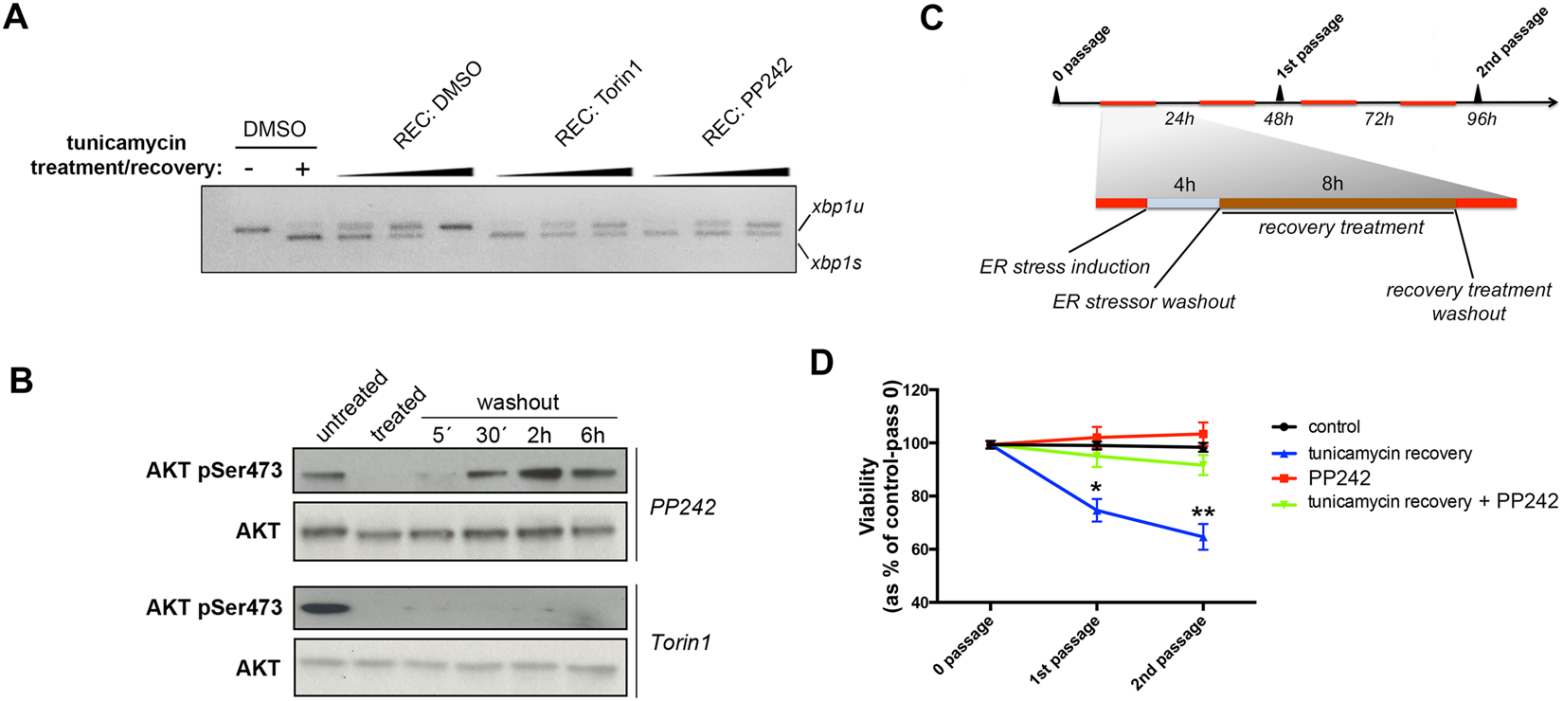
Delaying the attenuation of IRE1 RNAse activity affects cell fitness in the face of repeated, transient ER stress. (**A**) MCF10A cells were induced for ER stress with 500ng/ml tunicamycin for 4 h; washed out and allowed to recover either on vehicle-containing fresh medium (DMSO), PP242-containing medium (500 nM) or Torin1-containing medium (250 nM), and extracted for total mRNA for semiquantitative RT-PCR for the relative amounts of XBP1 mRNA species. (**B**) MCF10A cells were treated as indicated (PP242: 500 nM; Torin1: 250 nM), and whole cell lysates were analyzed for the levels of phosphorylated AKT or total AKT, as a readout of net TOR kinase activity. (**C**) A schematic depiction of the treatment regime is shown. MCF10A cells were exposed to 500ng/ml tunicamycin for 4 h to induce ER stress, then washed and allowed to recover for 8 h in fresh medium containing vehicle (DMSO) or mTOR kinase inhibitor (PP242; 500 nM), and further washed out and cultured for 12 h until the next treatment round. (**D**) 6 independent biological replicates were analyzed. Cells were trypsinized and resuspended in equal volumes, and counted using an automated live cell counter (Countess, Invitrogen).

In order to determine whether prolonged IRE1 activation following the transient engagement of ER stress (i.e. due to inhibition of AKT-mTOR signaling) could have long-term consequences, we devised an experimental regime to test whether recursive disruption of AKT-mTOR-dependent signaling during repeated rounds of ER stress affected cell fitness. To minimize effects on cell fitness by inhibition of mTOR independently from ER stress, we used a reversible mTOR kinase inhibitor, PP242, as opposed to the irreversible TOR kinase inhibitor Torin1^42^. Like Torin1, PP242 inhibits mTOR and prolongs IRE1 RNase activity, but its effect on the AKT-mTOR pathway is fully reversible upon washout of the inhibitor within 10 min (Fig. 5A and B). Thus we measured cell proliferation over two cell passages, during which cells were subjected to two rounds of acute, transient (4 h) ER stress induction, followed by a period of recovery in the absence or presence of transient mTOR inhibition (Fig. 5C). Repeated PP242 exposure and washout in the absence of ER stress did not have a detrimental effect on cell proliferation as compared to wild type cells (Fig. 5D). Recursive exposure to ER stress and washout diminished cell number overtime (Fig. 5D). In contrast, transient mTOR inhibition by exposure to PP242 during the periods of ER stress washout and recovery rescued the defects in cell fitness associated with repeated rounds of ER stress (Fig. 5D). These results suggest that mTOR inhibition prolongs IRE1 RNAse activity, which increases ER capacity, and over time, increases cell fitness.

### Transient IRE1 autophosphorylation is required for subsequent attenuation to take place

**Figure 6:**
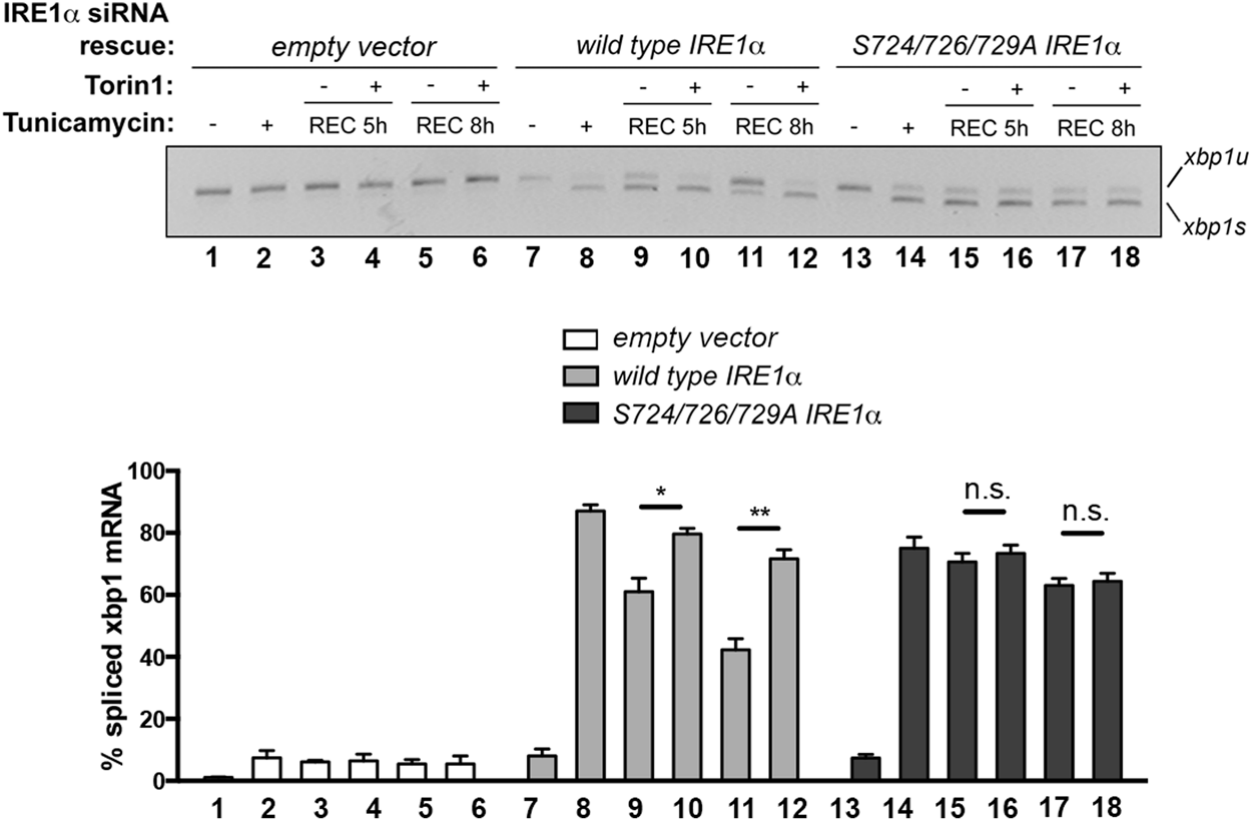
IRE1 KAL transient autophosphorylation is required for AKT-mTOR-dependent attenuation of IRE1 RNAse activity. Comparison of activity dynamics and Torin1 sensitivity between IRE1 wild type- and IRE1 S724/726/729A–expressing MCF10A, simultaneously silenced for endogenous IRE1, regarding their recovery from ER stress. Cells were sequentially transfected with IRE1 3′UTR-targeting siRNA, and siRNA-resistant cDNAs encoding either wild type IRE1 protein or a mutant version (see M&Ms and Figure S2). After the indicated treatments, total RNA samples were harvested and processed for RT-PCR analysis of XBP1 mRNA species. Data was derived from three independent biological replicates.

The autophosphorylation of residues within the KAL of IRE1 correlates with the dynamics of RNAse activity both in yeast^17,18^ and mammalian cells^15,36^. Based on previous studies^17,18,20,43^, we hypothesized that the transient autophosphorylation of the KAL domain of IRE1 regulates the deactivation of its RNAse domain, but is not strictly required for initial RNAse activity triggering. To compare the IRE1 attenuation dynamics between the wild type protein, and a non-phosphorylatable mutant, in MCF10A human epithelial cells, we used 3′ UTR-targeting siRNA to deplete endogenous IRE1 protein, while expressing either the wild type (WT) IRE1 protein, or a mutant version impaired for phosphorylation on the KAL (S724/726/729 A) from siRNA-insensitive minigenes (Figure S3A and B). Silencing of endogenous IRE1 is efficient using the 3′ UTR-targeting sequence (Fig. 6A, lanes 1–6; and Figure S3B). Of note, overexpressed wild type IRE1 efficiently spliced XBP1 mRNA in response to tunicamycin treatment, and its activity was attenuated upon removal of the source of ER stress (Fig. 6A, lanes 7–12; see Fig. 1C). mTOR inhibition also prolonged IRE1 RNAse activity in cells overexpressing wild type IRE1 (Fig. 6A, lanes 7–12). However, while the triple mutant version of IRE1 was capable of splicing XBP1 mRNA after tunicamycin treatment, this activity was markedly prolonged as compared to the RNAse activity of wild-type IRE1 following washout of the stressor agent (Fig. 6A). Furthermore, unlike the wild-type IRE1, the RNAse activity of the IRE1 triple mutant following washout is insensitive to mTOR inhibition (Fig. 6A, lanes 13–18). Notably, we recapitulated these results in *Ire1 −/−* mouse embryonic fibroblasts (MEFs) expressing either wild type or the triple mutant IRE1 cDNAs (see Figure S3C). These results further support a model by which the activation of the mammalian IRE1 RNAse domain does not strictly require IRE1 phosphorylation^43,44^, and that transient phosphorylation at the KAL of mammalian IRE1 kinase domain initiates the termination of IRE1 RNAse activity. Of note, competency on transient autophosphorylation also correlates with the functional impact of modulating IRE1 dynamics through AKT-mTOR signaling, because *Ire1* −/− MEFs stably expressing a non-phosphorylatable IRE1 mutant have similar fitness either in the absence or presence of AKT-TOR inhibition (Figure S4).

### AKT-mTOR signaling attenuates IRE1 RNAse activity by promoting ER-mitochondria contacts

**Figure 7:**
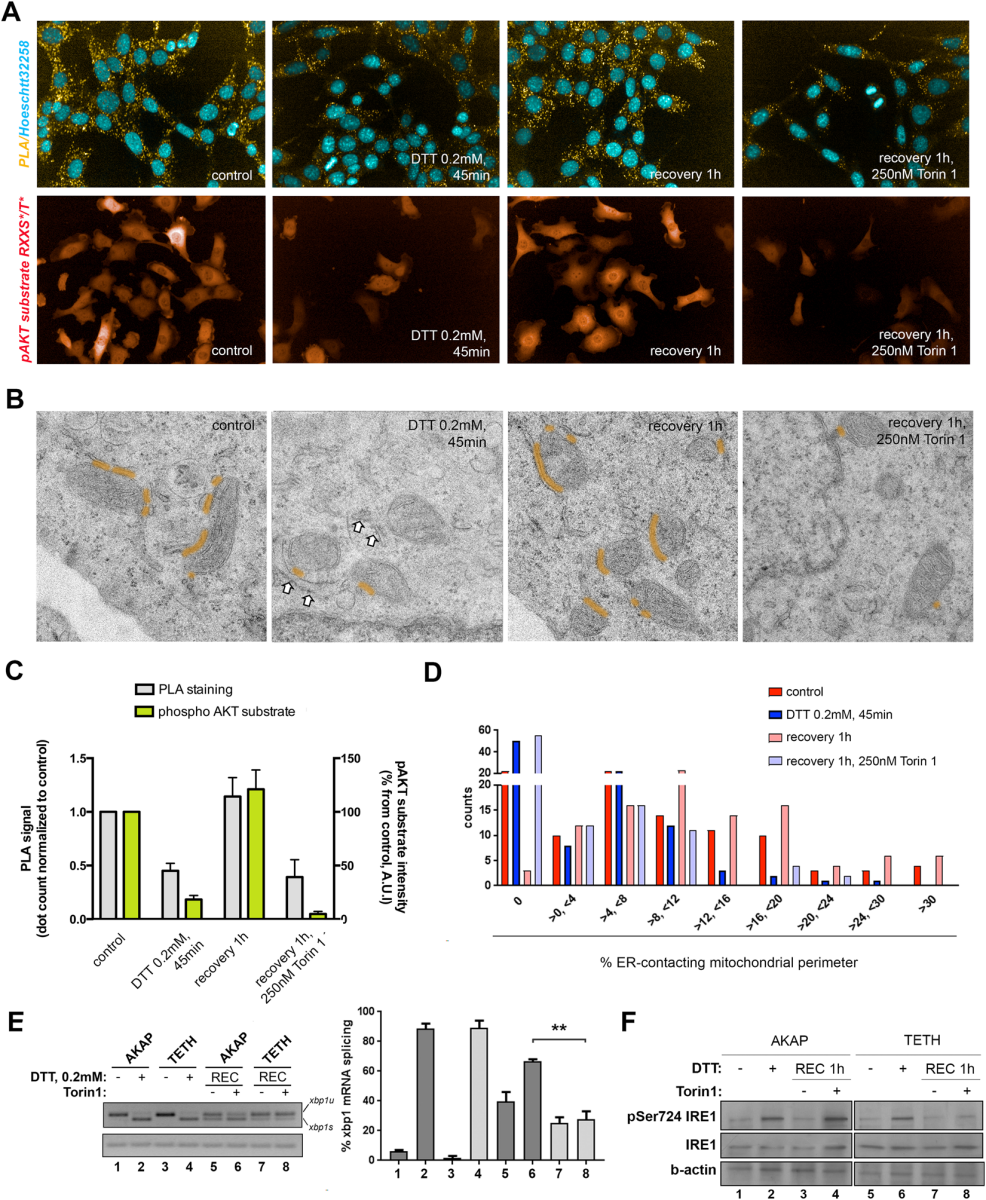
mTOR regulates the formation of ER-mitochondrial contacts which regulate IRE1 dephosphorylation and the attenuation of IRE1 RNAse activity. (**A**) MCF10A cells were either assayed for PLA-based IP3R-I-VDAC1 interaction extension (upper panel row) or phosphorylated AKT substrate (lower panel row) across a prototypical ER stress challenge and recovery assay. (*i*) untreated cells; (*ii*) cells challenged to ER stress for 45 min; (*iii*) cells challenged and allowed to recover in normal conditions; and (*iv*) cells challenged and allowed to recover in the presence of 250μM Torin1. (B) Representative electron microscopy images (12500x) of MCF10A cells as indicated. Orange hue tracks indicate regions of ER-mitochondria contact (<50 nm at widest gap). White arrowheads on the image corresponding to acute DTT exposure indicate aberrant, dilated sections of ER, which appear “detached” (>50 nm at narrowest width) from near-by mitochondria. (**C**) Quantitation of ~2000 cells per condition and label as shown in (**A**). (**D**) Quantitation of 100 mitochondria per condition for relative contact extension distribution from the image collections sampled in (**B**). (**E** and **F**) MCF10A cells either transduced with the control AKAP construct or the artificial ER-mitochondria tetherer (TETH) were treated as indicated, and either RNA (**E**) or protein (**F**) were assayed for relative XBP1 splicing levels and IRE1 phosphorylation, respectively.

Contacts between the ER and mitochondria at MAMs have been shown to be both regulated by, and regulate, AKT-TOR signaling: insulin promotes ER-mitochondrial contacts by promoting MAM stabilization/extension, and MAM abrogation results in reduced AKT-mTORC2 activity and insulin signaling^27,29^. Importantly, IRE1 might be also be regulated at MAMs^23^. Thus we hypothesized that a potential mechanism through which AKT-mTOR is regulating IRE1 RNAse dynamics is by promoting ER-mitochondria contacts. To test this model, we used a quantitative proximity ligation assay (PLA) assay to monitor the proximity of the ER and mitochondria in single cells, visualizing close pairs of the Inositol 3-P receptor I (IP3R-I) and the Voltage-dependent anion-selective channel 1 (VDAC1), which occur very specifically at ER-mitochondria contact sites (Figure S5A and^29^). According to previous reports, we confirmed that the PLA-based system can detect contacts between ER and mitochondria, the numbers of which are drastically reduced upon exposure to high concentrations of palmitate, as well as disruption of the AKT-mTOR pathway by Torin 1 exposure (Figure S5B; data not shown; and^27,29^). Importantly, we observed a pronounced reduction of PLA signal in cells acutely exposed to the ER stressor DTT (Fig. 7A and C), which was quickly recovered upon removal of the source of ER stress (Fig. 7A and C). Thus ER stress disrupts ER-mitochondria contacts, consistent with the fact that palmitate, which provokes sustained ER stress, is associated with MAM decrease^29,31^ (Figure S5B).

Torin1 exposure during ER stress recovery also impaired the reformation ER-mitochondrial contacts following washout of ER stressor (Fig. 7A and C). In accordance with a significant role of AKT-mTOR recovery dynamics for MAM recovery, the relative amount of phosphorylated AKT substrates (a proxy for AKT activity) correlates with formation/disruption of ER-mitochondria contacts (Fig. 7A and C). Taken together, these results support that ER-mitochondria contacts are disrupted during ER stress, concomitant with decreases in AKT kinase activity, and increases in IRE1 RNAse activity. As cells recover from ER stress, ER-mitochondria contacts reform in a AKT-mTOR-dependent manner that parallels the attenuation of IRE1 RNase activity. Specific disruption of mTORC1 activity during recovery from ER stress by exposure to rapamycin also exerted a delay on the reestablishment of ER-mitochondria contacts (Figure S5C).

We decided to conduct ultrastructural studies to validate the observations obtained using the PLA approach. We subjected MCF10A epithelial cells to the same treatment regimes described above, and fixed and processed them for electron microscopy. At the ultrastructural level, MCF10A cells exhibit prominent ER-mitochondria communication (apposition points below 50 nm width) in normal culture conditions, which are significantly abrogated upon acute exposure to ER stress (Fig. 7B; compare first two panels). In fact, certain features further supported such remodeling of the ER and mitochondria networks: we observed aberrant, enlarged ER cisterns, largely stripped from ribosomes, standing as retired from neighbouring mitochondria, which appeared fragmented (Fig. 7B; second panel from the left, arrowheads). Importantly, washout of the ER stressor led to restoration of ER-mitochondria communication, which was largely precluded by concomitant exposure to the TOR inhibitor Torin1 (Fig. 7B, right most panels).

To determine if suppression of IRE1 RNase activity was dependent on, and not simply correlated with, the AKT-mTOR-dependent reformation of stable ER-mitochondria contacts, we tested whether artificial stabilization of ER-mitochondria contacts could rescue the impaired attenuation of IRE1 RNAse activity that ensues upon AKT-mTOR inhibition. For this purpose, we generated lentiviral vectors expressing either an isolate peptide with high affinity for the outer mitochondrial membrane (OMM), or a tandem construct with this OMM-binding domain fused to an ER-targeting peptide (Figure S5D)- these constructs are hereon termed ‘AKAP’ and ‘TETH’ respectively. Analogous constructs have been successfully used to force or stabilize ER-mitochondria contact in a variety of experimental *in vitro* and *in vivo* models^45,46^. Of note, such artificial tethering stabilizes *functional* MAMs, because it recapitulates phenotypes associated with, and dependent on, effective ER-mitochondria tethering^45,47^. These constructs can be traced for appropriate subcellular localization and non-aberrant aggregation, by confocal fluorescence imaging, as they are fused to mRFP (Figure S5D^45^).

We assessed the impact of artificially tethering the ER to the mitochondria on the regulatory dynamics of IRE1 RNAase activity during recovery from ER stress. Removal of the ER stressor led to attenuation of IRE1 RNAse activity in AKAP-expressing cells, which was delayed upon exposure to Torin 1 as expected (Fig. 7F, lane 5 versus lane 6). IRE1 RNAse activity also was attenuated in TETH-expressing cells upon washout of the stressor agent (Fig. 7E, lane 7). However, mTOR inhibition did not delay the attenuation of IRE1 in TETH-expressing cells (Fig. 7F, lane 8). Thus artificially tethering the ER to the mitochondria bypasses the requirement for mTOR in the attenuation IRE1 RNAase activity during recovery from ER stress. To further assess the direct impact of manipulating ER-mitochondria contacts on IRE1 recovery, we assessed the effect of intervening a *bona fide* endogenous ER-mitochondria tether: phospho-furin acidic cluster sorting protein 2 (PACS2)^45^. RNAi-mediated depletion of PACS2 destabilizes ER-mitochondria contacts in MCF10A cells (Figure S5D). We measured the dynamics of IRE1 activation and deactivation in PACS2-knock down cells as compared with those of mock-transduced cells (Figure S5E). Importantly, PACS2-depleted cells exhibited blunted IRE1 attenuation as compared with control cells (Figure S5E), further supporting a role for the regulation of ER-mitochondria apposition for the negative control of UPR dynamics during ER stress recovery^45^.

**Figure 8:**
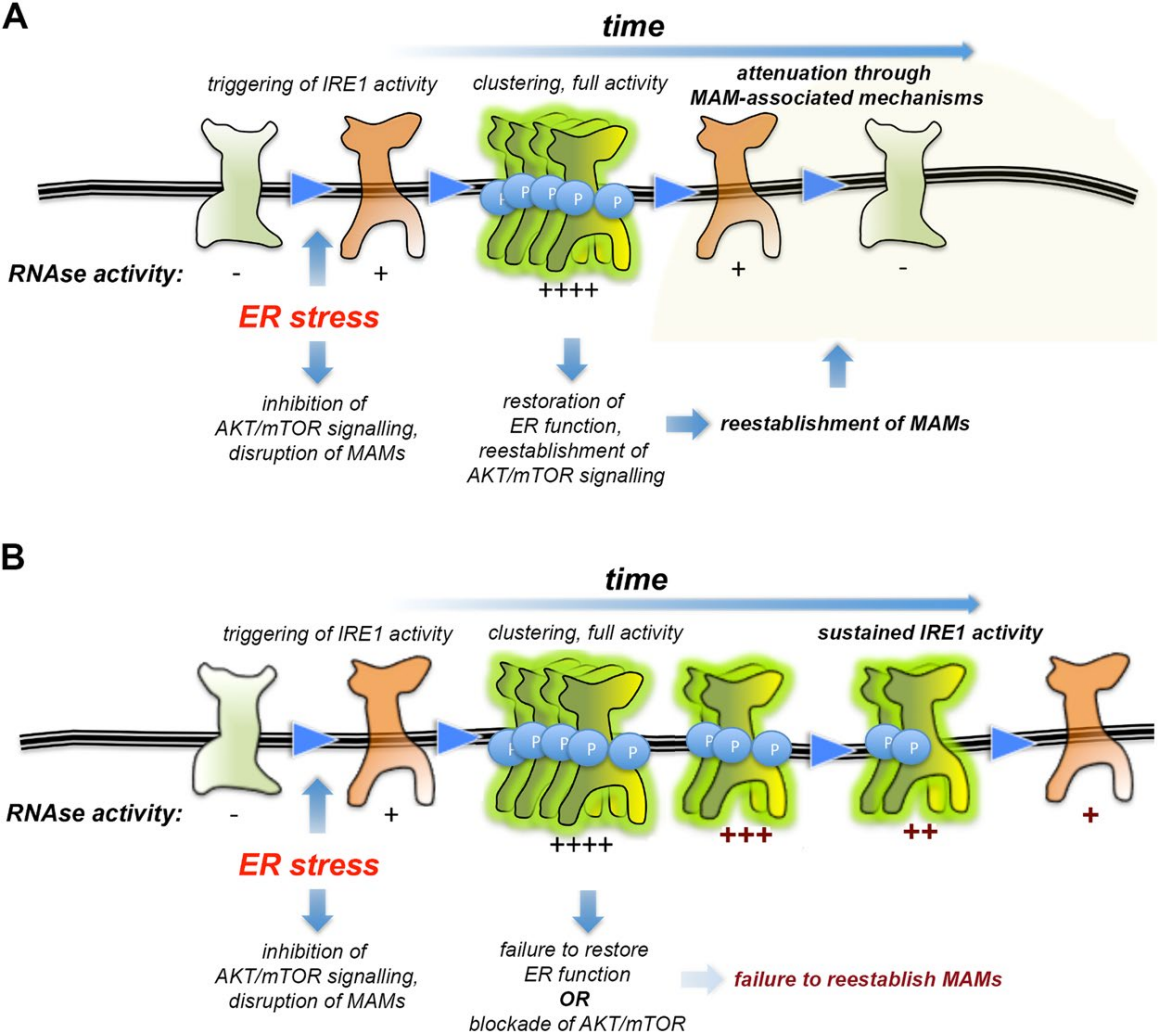
A model for AKT-mTOR-dependent modulation of IRE1 RNAse activity dynamics through regulating ER-mitochondria contacts. Depiction of events and proposed regulatory components for IRE1 RNAse activity dynamics when reactivation of AKT-mTOR signaling is enabled (**A**) or blocked (**B**). (**A**) Acute transient ER stress triggers IRE1 hyperphosphorylation and activity while temporarily shutting down AKT-mTOR signaling. Resolution of ER imbalance enables reconstitution of AKT-mTOR throughput, which favours ER-mitochondria communication and IRE1 shutdown. (**B**) When AKT-mTOR signaling recovery is impaired, independently of ER imbalance resolution, IRE1-attenuating mechanisms associated with ER-mitochondria contacts are abrogated, and IRE1 activity is extended in time.

To determine if ER-mitochondria contacts regulate IRE1 autophosphorylation that initiates the termination of RNase activity, we monitored phosphorylation of pSer724 in the KAL of IRE1 in AKAP or TETH expressing cells recovering from ER stress in the absence/presence of Torin. pSer724 was robustly phosphorylated when AKAP cells were exposed to ER stressor, and remained phosphorylated in the presence of Torin1 correlating with sustained levels of IRE1 RNAse activity (Fig. 7F). Phosphorylation of pSer274 also occured during ER stress when the ER was artificially tethered to the mitochondria. However, IRE1 was rapidly dephosphorylated either in the absence or presence of Torin1 during recovery from ER stress in TETH expressing cells. We interpret that in the absence of ER-mitochondria contacts, autophosphorylated IRE1 is not targeted properly for subsequent dephosphorylation and attenuation of IRE1 RNAse activity (Fig. 8).

## Discussion

We show that attenuation of IRE1 activity involves a two-step mechanism, where autophosphorylation first initiates termination of IRE1 RNAse activity, but cessation of RNAse activity only occurs if ER-mitochondria contacts are reformed after their initial uncoupling by ER stress. AKT-mTOR is one signaling axis which drives the formation of such contacts during recovery from ER stress. Thus, IRE1 phosphorylation dynamics appear to be a component of a ‘self-timing’ mechanism, where IRE1 autophosphorylation initiates the attenuation of IRE1 RNAse activity- but is not required for IRE1 activation itself and is not sufficient for inactivation *per se*, requiring its coordination with other homeostasis-determined events such as restoration of growth signaling and organelle communication. In engineering systems, ‘*self-destruct*’ mechanisms are often present where system malfunctions could lead to catastrophic consequences. Analogously, we propose that mechanisms to deactivate IRE1 RNAase activity are initiated immediately following engagement of the UPR, to limit prolonged IRE1 activity that could lead to damaging inflammation or apoptosis.

Our data is remarkably consistent with previous work in yeast^17,18,48^ and mammalian systems^19,36,43,49^, and supports the notion that IRE1 autophosphorylation is not a strict requirement for RNAse activation. Rather, once phosphorylation on the KAL has occurred, and the termination of RNAse activity has been presumably initiated, the KAL is dephosphorylated with kinetics similar to that of deactivation of the RNAse domain. In yeast, neither IRE1 mutations mimicking KAL constitutive phosphorylation, nor those rendering IRE1 unable to transiently autophosphorylate, abolish ER stress sensing, clustering, or RNase activity of IRE1. Instead, these mutations result in delayed deactivation of IRE1 RNase activity, and reduced adaptation to sustained ER stress^17,18,48^. We show that AKT-mTOR signaling regulates the attenuation of RNAse activity once autophosphorylation has occurred.

How AKT-mTOR-dependent stabilization of ER-mitochondria contacts favours deactivation of the IRE1 RNAse is unclear, but it might involve additional posttranslational modifications. For example, the red/ox state of specific key residues in IRE1 have been shown to alter its RNAse activity dynamics^21^. By promoting ER-mitochondria contacts, AKT-mTOR could gather autophosphorylated IRE1 oligomers to the proximity of red/ox gauging and oxidative stress management complexes enriched at those interorganelle domains^26^ thus allowing for the transference of red/ox information from the later to the former. An additional, non-exclusive mechanism might involve the regulation of IRE1 autophosphorylation by lipids. IRE1 is sensitive to perturbations in ER membrane bilayer composition and fluidity, independently from its luminal misfolding-sensing domain^6,24^. MAMs are raft-like subdomains of the ER, enriched in cholesterol^50,51^, and by stabilizing MAM contacts, AKT-mTOR signaling could shift the prevailing membrane environment into which a majority of IRE1 molecules dwell, thus altering its activation and deactivation thresholds through as yet undetermined mechanisms. This model could partially explain the tight integration observed among ER homeostasis, insulin signaling, and lipidostasis^6,25,29,31,45,52^. A third mechanism potentially regulated through stabilization/destabilization of ER-mitochondria contacts could be the negative control of IRE1 exerted by specific serine/threonine phosphatases, such as PPM1L and PP2A^19,53^. Of note, those two phosphatases have been found by us and others as *bona fide* MAM constitutive components^50,54^ and preliminary observations suggest that AKT-mTOR signaling regulates the interaction of subsets of these regulators with IRE1 *in vitro* (Sanchez-Alvarez M, Wang Y. and del Pozo MA, unpublished observations).

Recent reports show that ER stress can positively regulate ER-mitochondria communication in HeLa cells^55,56^. In our experimental system, acute ER stress promoted a clear disruption of ER-mitochondria communication, and its AKT-mTOR-dependent re-establishment clearly correlated with the dynamics of UPR signaling. As demonstrated by previous studies on IRE1 shutdown dynamics in yeast^18^, mechanisms driving UPR dynamics are likely contextual, and determined by proliferation and metabolic specificities. In fact, we observe HeLa cells to be comparatively refractory to Torin1-mediated delay on UPR recovery, as compared with other cell lines (Figure S1C). An alternative possibility might rely on those studies actually reflecting initial recovery phases, where ER-mitochondria contacts are starting to be reestablished.

Our data supports a model whereby ER-mitochondria contacts are specialized signaling platforms, where several environmental stimuli and cellular conditions can be sensed simultaneously and integrated by protein and lipid components, thus enabling the orchestration and fine-tuning of multiple downstream responses. A rationale for such concentration of converging signals might partially derive from the fact that many input signals (especially lipids and poorly folded proteins) have very reduced diffusion, whereas others such as ROS, small metabolites, and ATP tend to rapidly diffuse from their sites of origin. By bringing the relevant signals into close proximity with each other allows maximal coincident detection and integration. Critically, signaling at ER-mitochondria contacts appears to feedback on the formation of the contacts themselves: for example, AKT-mTOR signaling, which requires intact MAM tethering components, promotes the formation of further contacts^27,29^. Intriguingly, dyslipidemic conditions associated with obesity, which provoke loss of ER homeostasis, also lead to altered ER-mitochondria communication, further suggesting a central role of the dynamical control of these structures in coping with metabolical and proteotoxic challenges^29,31,32,45,52^. An intriguing open question pertains as to whether mTORC1 or mTORC2 are specifically engaged to exert this regulation. In fact, mTORC2 and mTORC1 might be exerting their influence through independent, converging mechanisms, as has been proposed for another critical node for ER homeostasis- SREBP1 positive regulation^57^. Our observations support a specific role for mTORC1-dependent signaling in dictating these mechanisms, which might be derived from its positive control of phospholipid anabolism^31,58^. Future mechanistic studies may shed light onto the specific contribution of each signaling module in a context-specific manner, and their molecular dynamics across the kinetics of ER stress recovery.

AKT-mTOR mediated regulation of of IRE1 RNAse dynamics has relevant consequences for cellular homeostasis, as prolonged IRE1 activity leads to sustained ER expansion during ER stress recovery, and increased fitness over repeated rounds of acute ER stress. These observations are in line with recent work demonstrating that regulation of “short-term” signaling dynamics (i.e. on the order of minutes-hours) during the UPR can have long-term benefits to cell fitness, and potentially tissue and organismal homeostasis. For example, inhibition of the PPP15B phosphatase complex by the adrenergic agonist guanabenz disrupts negative feedback on PERK thus extending the activity of PERK branch of the UPR and promotes an increase in cellular fitness in the face of moderate ER stress^59,60^. An interesting hypothesis would be that AKT-mTOR ‘curb’ ER expansion during ER stress recovery, to accommodate protein anabolism with ER physical capacity. Such model would also partially explain why ER-mitochondria contacts (which in turn depend on ER architecture dynamics) have evolved as regulatory elements of this homeostatic system. Because aberrant UPR activation, uncompensated proteostasis, and dysregulated AKT-mTOR signaling are common drivers of major worldwide health threats, including type 2 diabetes and obesity-related syndromes, neurodegenerative diseases, and certain types of cancer such as hepatocellular carcinoma^3,61–67^, we propose that the AKT-mTOR-dependent IRE1 shutdown mechanism might constitute a novel avenue for therapeutic manipulation.

## Material and Methods

### Cell culture and reagents

S2R+ Drosophila cells (DRSC) were cultured in Schneider’s medium (Sigma) with 10% fetal bovine serum (Gibco). MCF10A (kindly provided by professor Clare Isacke, ICR, UK) were cultured in DMEM-F12 Glutamax medium (Gibco), supplemented with 5% horse inactivated serum (Sigma), 10ng/ml EGF (Preprotech), 25 μg/ml hydrocortisone (Sigma), recombinant insulin (Sigma) and 10U cholera toxin. 293T, HeLa and MDA-MB231 cells were grown on high glucose DMEM supplemented with 10% FBS, pen/strep, 1 mM pyruvate, 4 mM glutamine. *Ire1* −/− mouse embryonic fibroblasts (MEFs) were a kind gift from professor David Ron (Cambridge University, UK)^68^, and were cultured in high glucose DMEM supplemented with 10% FBS, 10 mM HEPES, pen/strep, 1 mM pyruvate, 4 mM glutamine, non-essential amino acids (Lonza), 0.2 mM HEPES pH7.6 (Lonza) and 400 mg/ml gentamycin (Gibco). Tunicamycin, rapamycin, tauroursodeoxycholic acid (TDCA), 4-phenylbutirate (4PBA), iodoacetamide and dithiothreitol (DTT) were purchased from Sigma; Torin1 and PP242 were purchased from Tocris. Clones from stable 293T cells transfected with the pcDNA5.1 IRE1-6His-3xFLAG-EGFP construct/FLP^36^ (kindly provided by professor Peter Walter, UCSF, USA) were selected after passage in DMEM supplemented with 10% FBS and 20 μg/ml hygromycin and sorting twice for fluorescence homogeneity, and tested for basal IRE1-EGFP activity, and relative expression level (see Figure S1). For *in vivo* IRE1 clustering dynamics experiments, optical 96-well plates were pre-coated with HMW poly-L-lysine (>300000 MW; Sigma) following standard procedures. After lentiviral transduction, clones with low expression levels were sorted and assessed for IRE1 protein expression. Propidium iodide was purchased from Sigma. Antibodies targeting pSer724 IRE1, PDI, BiP, VDAC1 and total eIF2alpha were obtained from Abcam; antibodies targeting pSer473AKT, pSer308AKT, total AKT, pSer2248 FRAP1 (TOR kinase), pThr33/37 4EBP1 and total 4EBP1, pSer235/236 S6, total S6 and total IRE1 were from Cell Signaling. Antibodies against GAPDH and tubulin were purchased from Novus. Anti-I3PR-I and ATF6 antibodies were purchased from SantaCruz Bt. and anti-pSer51eIF2alpha antibody was obtained from Enzo Biosciences Ltd. Anti-Hsp47 and anti-PACS2 antibodies were purchased from Human Protein Atlas consortium.

### Nucleotide transfections and RT-PCR proceedings

For transient knockdown, MCF10A cells were reverse transfected with Lipofectamine RNAiMAX following recommendations of the supplier (Invitrogen), with siRNA duplexes from Dharmacon. The 3′-UTR siRNA duplex used in the experiments described in Fig. 4C was custom-synthesized by Dharmacon and comprises the targeting sequence CTTCACTGGAGACCGGAATTG. In those experiments, the custom siRNA was reverse transfected at 120 nmol/15000 cells; 24 h later, cells were further transfected with 100 ng of the indicated pcDNA3.1-IRE1 constructs (Dr. Maruf Ali, Imperial College of London) using Lipofectamine 3000. In pilot experiments using an EGFP reporter driven from the same plasmid backbone, the % of positively DNA-transfected cells was >80%. ~48 h after plating, treatment courses were completed, and total RNA was extracted for the analysis of XBP1 mRNA species. S2R+ cells were reverse transfected where indicated with ~2 μg of dsRNA using Effectene (Qiagen) following previously described protocols^31^, and the DSRC amplicon for Tor was DRSC36734. The tethering constructs were engineered by PCR proceedings from the inducible tethering FRET constructs described in^47^ to be expressed from 2^nd^ generation lentiviral vectors (Viral Vector unit, CNIC). The lentiviral vector expressing an shRNA against PACS2 was purchased from Sigma (TRCN0000168619). Genomic DNA-free, total RNA samples, were prepared and processed for RT-PCR or qRT-PCR as previously described^31^. XBP1 splicing ratio was calculated as described [30]- briefly, gel densitometry was calculated on raw, unprocessed image captures and after custom background correction, [XBP1s/XBP1u + XBP1s] was computed for each sample. A list of the primer sequences used is provided as supplementary material.

### Automated imaging

Acapella Studio image analysis software was used for all procedures, on images acquired in an Opera QEHS station (PerkinElmer). All liquid handling and staining procedures were performed as previously described^31^ in an Opera Explorer II robotic station. For the assessment of clustering degree of IRE1, bright foci (>1.3 the average intensity of the cell) were segmented, and the relative contribution to the whole intensity was estimated as a % of IRE1 in clusters^36^. For the assessment of relative ER spatial redistribution/expansion, subcellular zones (periphery and inner regions) were selected with boundaries as constant relative percentages of the total area, and the ratio of their average intensities for an ER-specific marker (PDI), once normalized to tubulin intensity, was calculated^31,32^.

Proximity ligation assay (PLA) protocols were based on a previous report^29^ and were carried out using a DuoLink Orange kit (Sigma). Positive interaction detection and intensity classification was equally carried out using the Acapella Studio platform (PerkinElmer).

### Electron microscopy

MCF10A cells grown on 100-mm dishes treated as indicated were fixed with 4% paraformaldehyde and 2% glutaraldehyde for 120 min at room temperature. Upon gentle scrapping, postfixation was carried out with 1% OsO_4_ and 1.0% K_3_Fe(CN)_6_ in H_2_O at 4 °C for 60 min. Samples were dehydrated with ethanol and embedded in Epoxy, TAAB 812 Resin (TAAB Laboratories) according to standard procedures. Ultrathin (80 nm) sections were stained with saturated uranyl acetate and lead citrate and visualized with a JEOL JEM 1010 (Tokyo, Japan) electron microscope at 80 kV. 16-bit images were recorded with a 4 k × 4 k CMOS F416 camera from TVIPS (Gauting, Germany), typically at 12000X magnification. 100 individual mitochondria were analyzed per condition, and their total perimeter and ER-contacting fraction were assessed upon manual segmentation using ImageJ.

### Protein analysis and *in vitro* analysis of ER luminal parameters

Most protein analyses were performed on whole cell extracts in conventional 10% reducing SDS-PAGE conditions unless otherwise stated^31^. Assessment of relative red/ox environment in the ER lumen was performed by non-reducing SDS polyacrylamide gel electrophoresis of whole cell extracts obtained following *in vivo* alkylation (20 mM iodoacetamide, ~1 min) and western blot analysis for endogenous species of PDI (adapted from^31,39^). Relative glycosylation of the Hsp47 chaperone was analyzed from whole cell lysates through conventional SDS-PAGE and western blot analysis in precast 4–20% Acrylamide-Bisacrylamide Bis-Tris gels (Invitrogen). High molecular weight (HMW) molecular aggregates containing the BiP chaperone were isolated by sucrose cushion separation from post-nuclear lysate supernatants, from material obtained from 2 * 10^7^ cells^39,60^.

## Electronic supplementary material

Supplementary Information
