# Publisher Correction: AKT-mTOR signaling modulates the dynamics of IRE1 RNAse activity by regulating ER-mitochondria contacts

**DOI:** 10.1038/s41598-018-24796-z

**Published:** 2018-04-19

**Authors:** Miguel Sanchez-Alvarez, Miguel Angel del Pozo, Chris Bakal

**Affiliations:** 1 Dynamical Cell Systems Team, Division of Cancer Biology, The Institute of Cancer Research-Chester Beatty laboratories, 237 Fulham Rd, SW3 6JB London, United Kingdom; 2 Mechanoadaptation and Caveolae Biology Lab, Area of Cell and Developmental Biology, National Centre for Cardiovascular Research (CNIC), c/Melchor Fernandez Almagro, 8, CP, 28029 Madrid, Spain

Correction to: *Scientific Reports* 10.1038/s41598-017-16662-1, published online 28 November 2017

There was an error in Figure 3A in the original Article, where the numbers below the blot were incorrectly duplicated. This error has now been corrected in the PDF and HTML versions of the Article.

**Figure 3:**
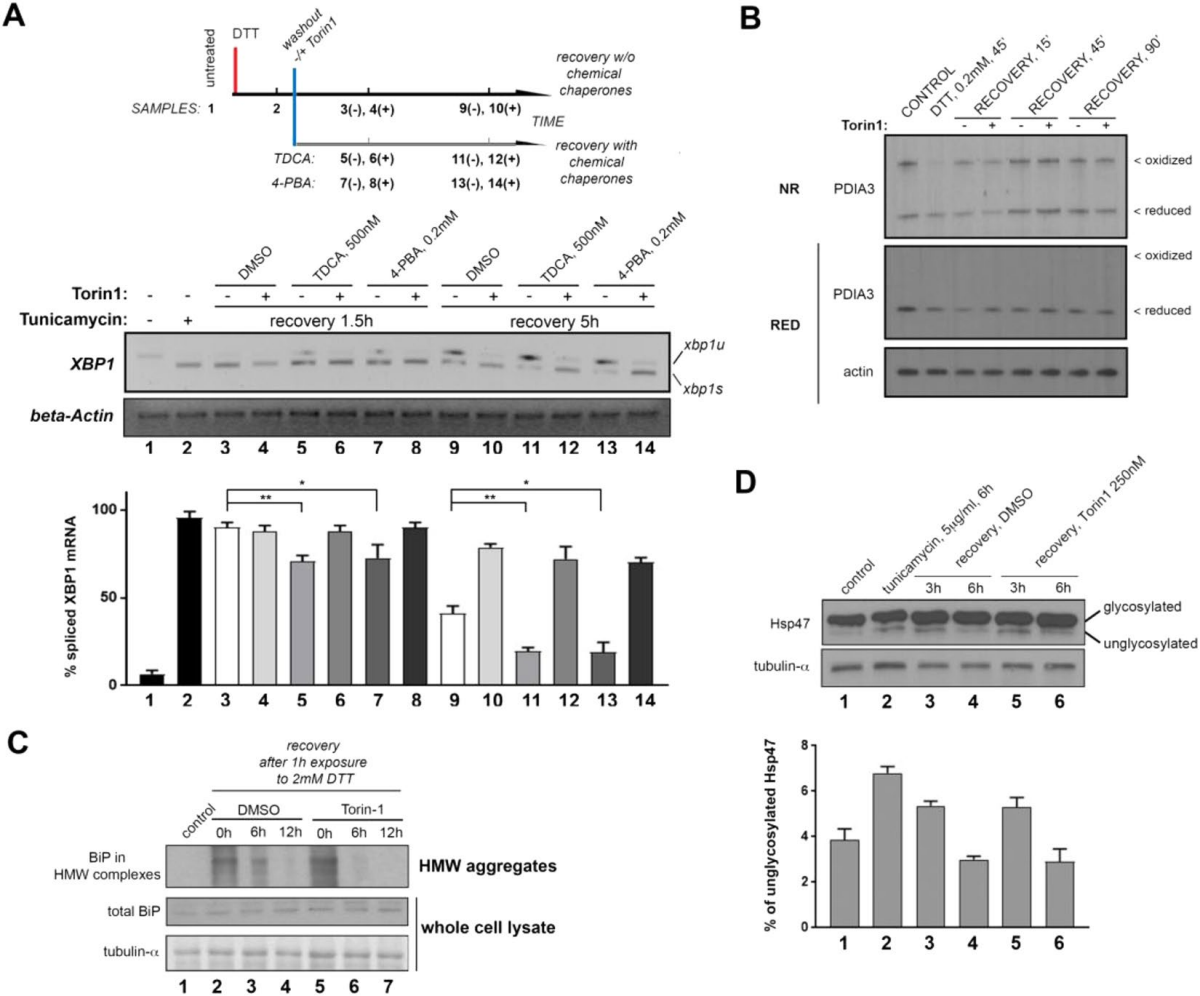
Inhibition of mTOR1 does not prolong IRE1 RNAse activity due to increases in ER stress. (**A**) Chemical chaperones TDCA or 4-PBA are not capable of reverting the delay in IRE1 attenuation induced by TOR kinase inhibition during ER stress recovery. TDCA: tauroursodeoxycholic acid; 4-PBA: 4-phenylbutiric acid. Cells were treated as indicated, and total RNA was extracted for RT-PCR analysis of XBP1 mRNA species. Data was derived from four experimental replicates. (**B**) Non-reducing SDS-PAGE analysis of endogenous species of PDIA3. MCF10A were treated as indicated (Torin1: 500 nM) and immediately alkylated and lysed in non-reducing sample buffer and analyzed by western blot analysis. Equal amounts of unalkylated sample were lysed in DTT-containing sample buffer and run in parallel for total PDIA3 levels. Oxidized species and reduced species are indicated in the upper panel (NR: non-reducing SDS-PAGE). (**C**) High molecular weight (HMW) aggregates from MCF10A cells either left untreated, or exposed to 2 mM DTT for 1 h and then allowed to recover in fresh media with the indicated treatments for the indicated times, were isolated by sucrose cushion centrifugation, solubilized in 8 M urea-sample buffer and analyzed by western blot (38) [top panel]. Unprocessed extracts were analyzed in parallel [middle and bottom panels]. (**D**) Whole cell lysates from MCF10A cells treated as indicated were resolved through gradient SDS-PAGE gels, and analyzed by western blot for species of the Hsp47 collagen chaperone. Glycosylated (top band) and hypoglycosylated (lower band) species are indicated in the corresponding western blot. Densitometry data is shown in the accompanying graph for three biological replicates.

