# Supplementary material for: AKT-mTOR signaling modulates the dynamics of IRE1 RNAse activity by regulating ER-mitochondria contacts
Source: Sci Rep. 2017 Nov 28;7:16497. doi: 10.1038/s41598-017-16662-1 (PMC5705697; doi:10.1038/s41598-017-16662-1)
Supplement: Supplementary file 1 — Supplementary Information [file 41598_2017_16662_MOESM1_ESM.pdf]

MANUSCRIPT SUPPLEMENTARY INFORMATION

AKT-mTOR signaling modulates the dynamics of IRE1 RNAse activity by regulating ER-mitochondria contacts

Miguel Sanchez-Alvarez, Miguel Angel del Pozo and Chris Bakal

CONTENT: 5 supplementary figures and legends

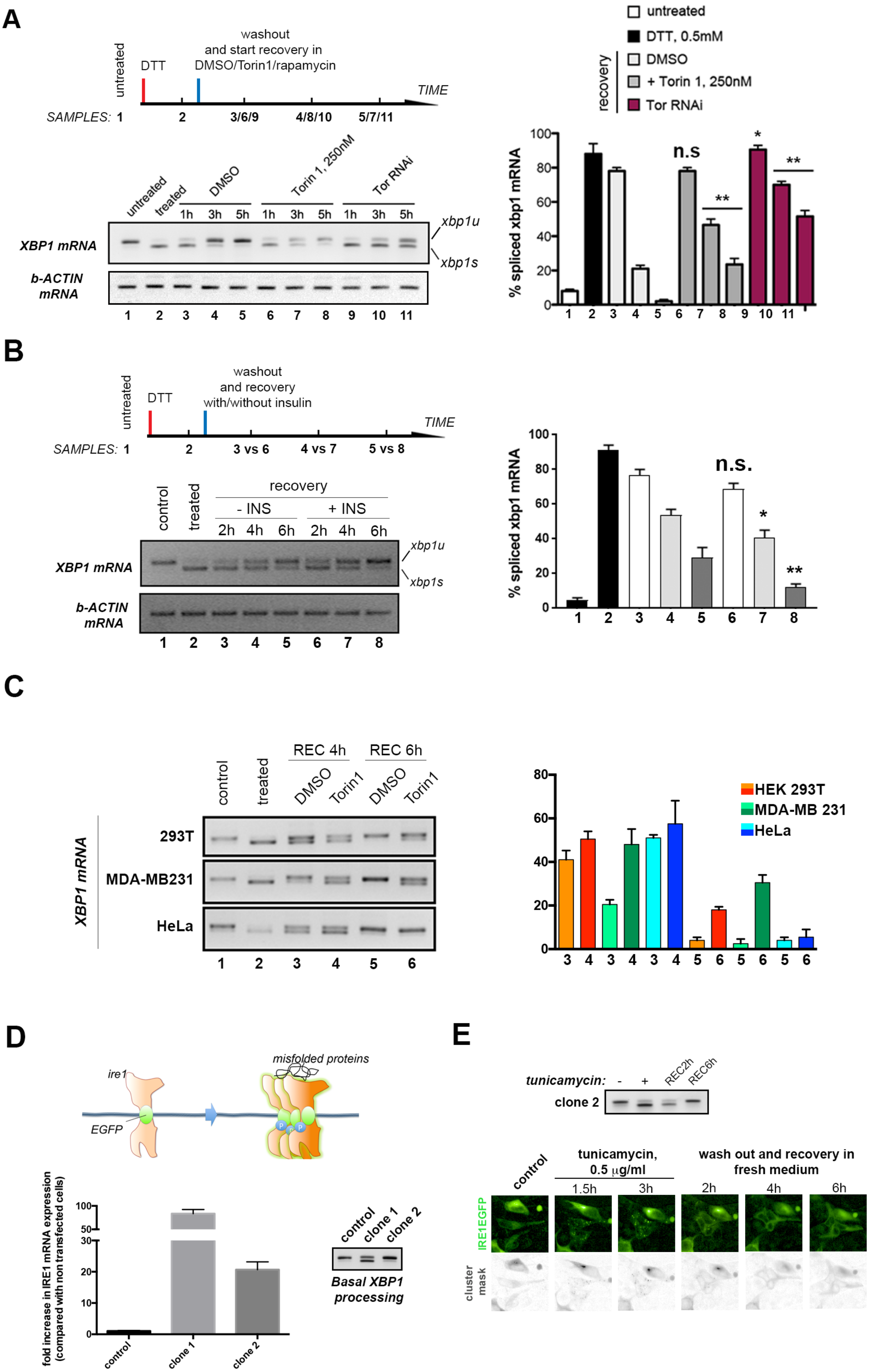
A
untreated
DTT
washout
and start recovery in
DMSO/Torin1/rapamycin
TIME
SAMPLES: 1
2
3/6/9
4/8/10
5/7/11
untreated
treated
DMSO
Torin 1, 250nM
Tor RNAi
1h 3h 5h 1h 3h 5h 1h 3h 5h
XBP1 mRNA
b-ACTIN mRNA
xbp1u
xbp1s
1 2 3 4 5 6 7 8 9 10 11
untreated
DTT, 0.5mM
recovery
DMSO
+ Torin 1, 250nM
Tor RNAi
% spliced xbp1 mRNA
n.s
**
*
**
B
untreated
DTT
washout
and recovery
with/without insulin
TIME
SAMPLES: 1
2
3 vs 6
4 vs 7
5 vs 8
control
treated
recovery
- INS
+ INS
2h 4h 6h 2h 4h 6h
XBP1 mRNA
b-ACTIN mRNA
xbp1u
xbp1s
1 2 3 4 5 6 7 8
% spliced xbp1 mRNA
n.s.
*
**
C
control
treated
REC 4h
REC 6h
DMSO
Torin1
DMSO
Torin1
XBP1 mRNA
293T
MDA-MB231
HeLa
1 2 3 4 5 6
HEK 293T
MDA-MB 231
HeLa
3 4 3 4 3 4 5 6 5 6 5 6
D
ire1
EGFP
misfolded proteins
fold increase in IRE1 mRNA expression
(compared with non transfected cells)
control
clone 1
clone 2
Basal XBP1 processing
E
tunicamycin: - + REC2h REC6h
clone 2
control
tunicamycin, 0.5 μg/ml
1.5h
3h
wash out and recovery in fresh medium
2h
4h
6h
IRE1EGFP
cluster mask

Figure S1: Supplementary information pertaining experiments presented in figure 1 (A) Tor RNAi (96h) in *Drosophila* S2R+ cells recapitulates the defects in IRE1 attenuation observed upon pharmacological inhibition of Tor. Total RNA samples from the indicated time points were extracted for RT-PCR analysis of XBP1 mRNA species. Data is derived from three independent biological replicates. (B) MCF10A cells were cultured following standard procedures and deprived of insulin for 18h. After ER stress stimulation (1h, 0.5mM DTT) recovery was allowed in the same insulin-deprived medium or medium supplemented with 20microg/ml insulin. Total RNA samples from the indicated time points were extracted for RT-PCR analysis of XBP1 mRNA species. Data was derived from three independent biological replicates. (C) Different cell lines were subjected to analogous ER stress recovery analyses as previously described. Total RNA samples from the indicated time points were extracted for RT-PCR analysis of XBP1 mRNA species. Data was derived from three independent biological replicates. (D) The IRE1EGFPv2 clone exhibits reversible IRE1 activity comparable to that of wild type cells, which is in turn paralleled by spatial clustering dynamics. Analogous time courses of acute ER stress induction and subsequent washout and recovery were monitored either by RTPCR analysis of XBP1 mRNA species and IRE1 spatial clustering. A grey inverted intensity mask showing IRE1 foci is overlayed.

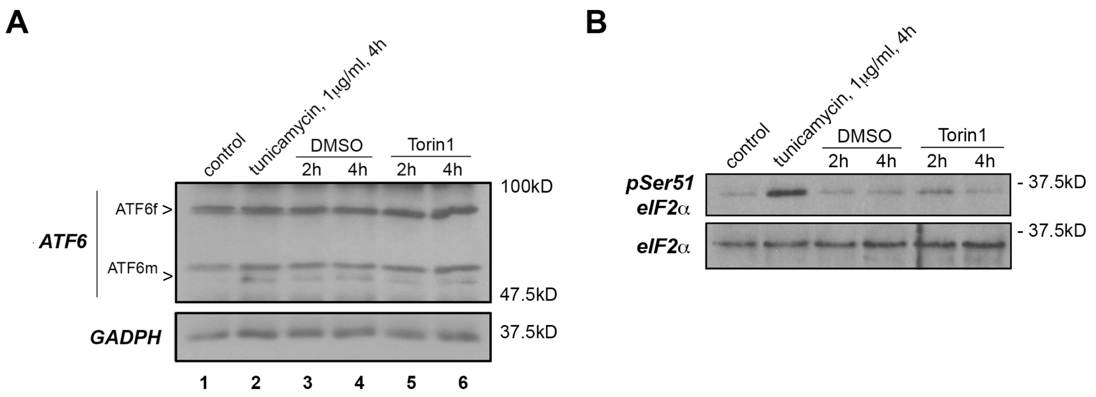

Supplementary figure 2.- TOR kinase inhibition does not significantly impact the attenuation dynamics of the other two main branches of the UPR. (A) MCF10A cells were treated as indicated, and whole cell lysates were analyzed by western blot for relative levels of full length [upper panel] or cleaved ATF6 species [lower panel]. Relative semiquantitation of two independent biological replicates is shown. (B) Analogous experiment is shown analyzing the main downstream target of the EIF2AK3/PERK kinase.

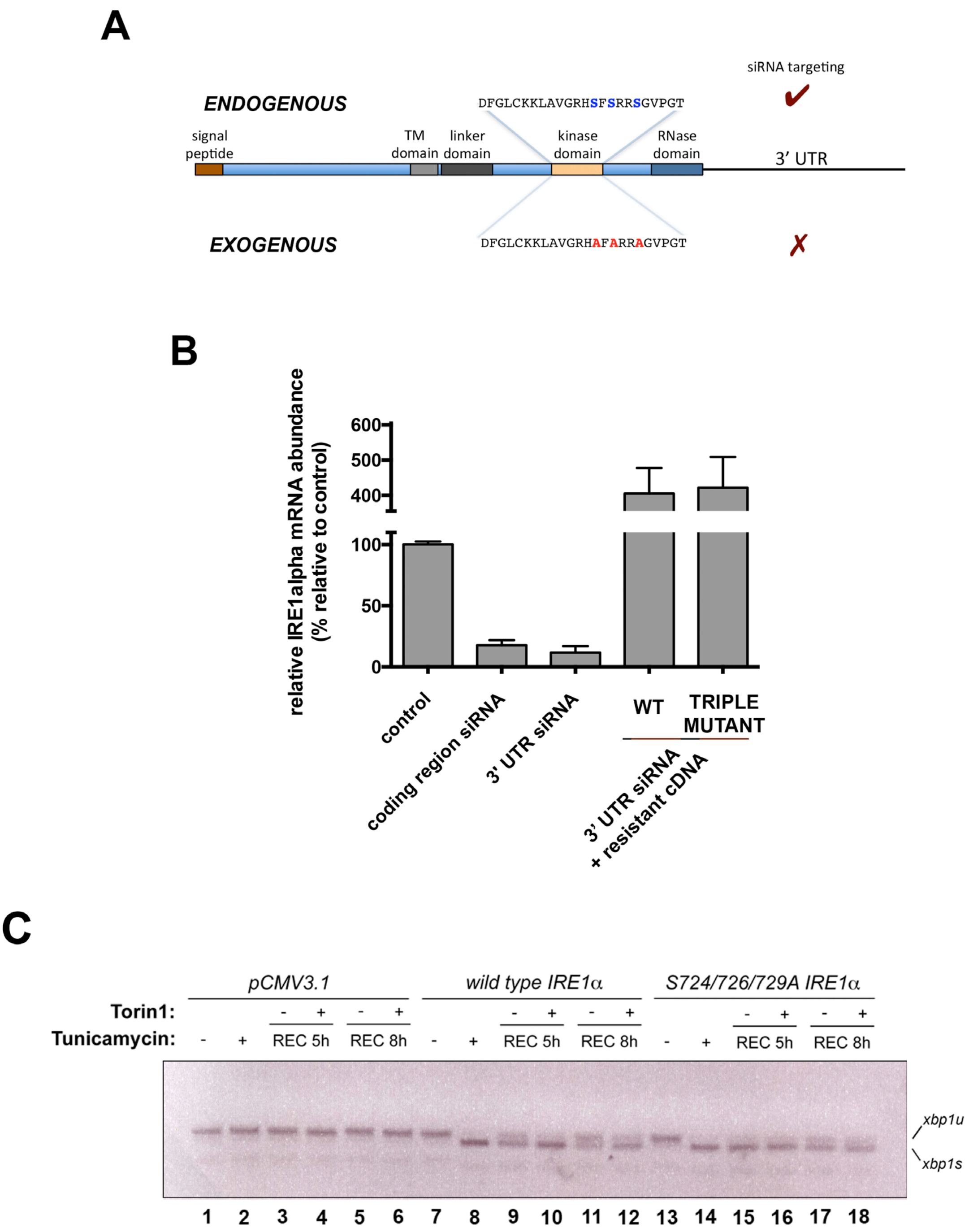

Supplementary figure 3.- Outline and validation of the strategy used to study the activity dynamics of IRE1 wild type and mutant versions from minigenes in MCF10A cells (A) Outline of the experiments and specification of the IRE1 KAL mutant sequence used in MCF10A cells. (B) qRTPCR for IRE1 mRNA species from MCF10A cell samples samples across treatments, including cDNA-rescuing conditions in an 3'UTR siRNA silencing background. Graphs comprise three biological triplicates. (C) *Ire1* -/- MEFs transduced with the indicated expression constructs were assayed for an analogous experimental regime as detailed in fig. 4 and analysis of XBP1 splicing.

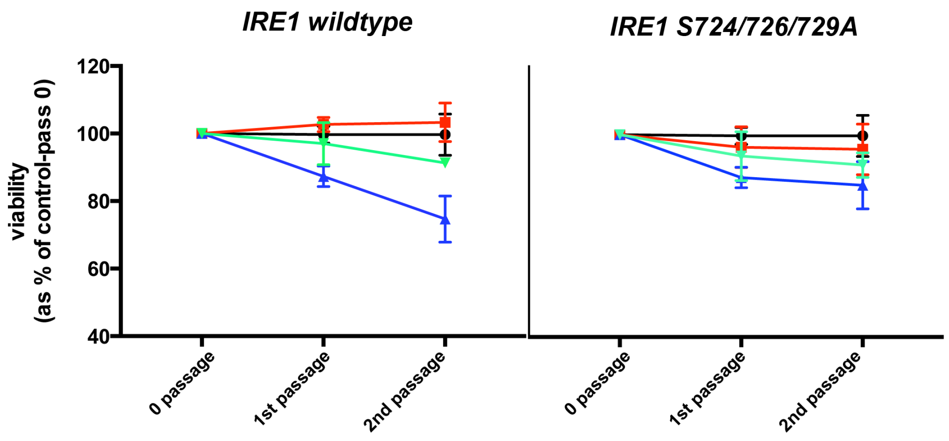

Supplementary figure 4.- (referred to figure 5) analysis of cell fitness upon recursive exposure to transient ER stress. Cells from either background were subjected to the treatment regimes exposed in figure 5. Four independent biological replicates were analyzed.

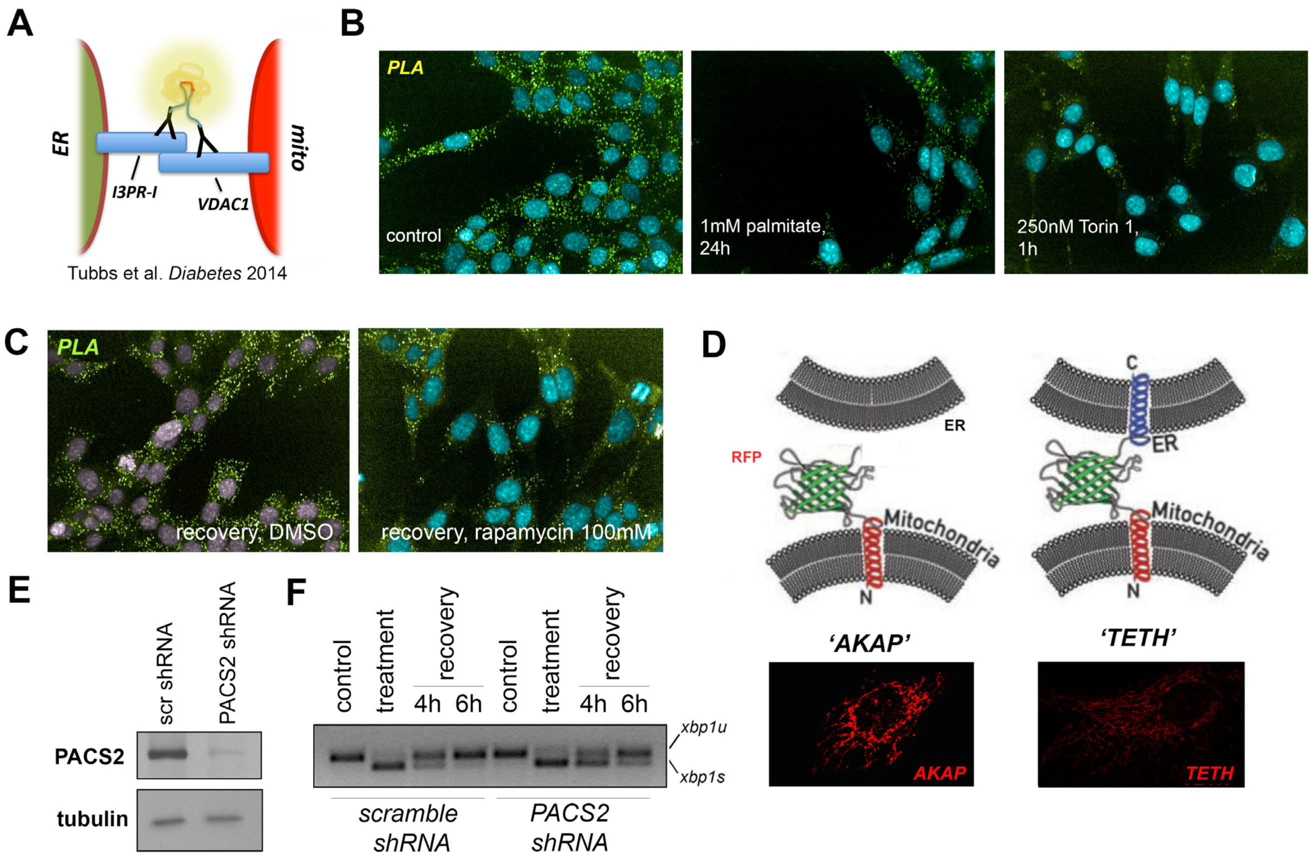

Supplementary figure 5.- (A) Schematic depiction of the basis of the PLA assay as designed for the detection of ER-mitochondria apposition (see ref. [28]). (B) PLA staining is specific and we recapitulate a decreased in the density of MAM contacts upon treatment with 1mM palmitate, 24h [28] (center panel). As predicted from the high dependency of MAMs on insulin signaling, exposure to the mTOR inhibitor Torin1 significantly reduces MAM-associated PLA signal. (C) Exposure to rapamycin during ER stress recovery decreases PLA signal in MCF10A cells. MCF10A cells were exposed to 0.2mM DTT for 45min, and washed to recover for 1h in in fresh medium supplemented either with DMSO or 100nM rapamycin. Cells were then fixed and processed for PLA staining. ~2000 cells were imaged per condition. (D) Schematic depiction of the AKAP and TETH synthetic constructs. Direct imaging of the AKAP and TETH synthetic constructs (mRFP imaging) is shown in the lower panels. (E and F) Disruption of ER-mitochondria contacts through shRNA-mediated knockdown of the MAM scaffold PACS2 is associated with delayed IRE1 attenuation.
